# Use of Spectroscopic Techniques to Monitor Changes in Food Quality during Application of Natural Preservatives: A Review

**DOI:** 10.3390/antiox9090882

**Published:** 2020-09-17

**Authors:** Abdo Hassoun, Maria Carpena, Miguel A. Prieto, Jesus Simal-Gandara, Fatih Özogul, Yeşim Özogul, Özlem Emir Çoban, María Guðjónsdóttir, Francisco J. Barba, Francisco J. Marti-Quijal, Anet Režek Jambrak, Nadica Maltar-Strmečki, Jasenka Gajdoš Kljusurić, Joe M. Regenstein

**Affiliations:** 1 Nofima AS, Norwegian Institute of Food, Fisheries, and Aquaculture Research, 9291 Tromsø, Norway; 2 Nutrition and Bromatology Group, Department of Analytical and Food Chemistry, Faculty of Food Science and Technology, University of Vigo, Ourense Campus, 32004 Ourense, Spain; maria.carpena.rodriguez@uvigo.es (M.C.); mprieto@uvigo.es (M.A.P.); jsimal@uvigo.es (J.S.-G.); 3 Department of Seafood Processing Technology, Faculty of Fisheries, Cukurova University, Adana 01330, Turkey; fozogul@cu.edu.tr (F.Ö.); yozogul@cu.edu.tr (Y.Ö.); 4 Fisheries Faculty, Fırat University, Elazığ 23200, Turkey; oecoban@firat.edu.tr; 5 Faculty of Food Science and Nutrition, University of Iceland, 113 Reykjavík, Iceland; mariagu@hi.is; 6 Matis, Food and Biotech R&D, 113 Reykjavík, Iceland; 7 Nutrition and Food Science Area, Preventive Medicine and Public Health, Food Science, Toxicology and Forensic Medicine Department, Faculty of Pharmacy, Universitat de València, 46100 València, Spain; francisco.barba@uv.es (F.J.B.); francisco.j.marti@uv.es (F.J.M.-Q.); 8 Faculty of Food Technology and Biotechnology, University of Zagreb, 10 000 Zagreb, Croatia; Arezek@pbf.hr (A.R.J.); jgajdos@pbf.hr (J.G.K.); 9 Ruđer Bošković Institute, Division of Physical Chemistry, Bijenička c. 54, 10 000 Zagreb, Croatia; nstrm@irb.hr; 10 Department of Food Science, Cornell University, Ithaca, NY 14853-7201, USA; jmr9@cornell.edu

**Keywords:** essential oils, fluorescence, UV-Vis spectroscopy, Fourier transform infrared, Raman, edible films, shelf-life, antioxidant, antimicrobial

## Abstract

Consumer demand for food of high quality has driven research for alternative methods of food preservation on the one hand, and the development of new and rapid quality assessment techniques on the other hand. Recently, there has been a growing need and interest in healthier food products, which has led to an increased interest in natural preservatives, such as essential oils, plant extracts, and edible films and coatings. Several studies have shown the potential of using biopreservation, natural antimicrobials, and antioxidant agents in place of other processing and preservation techniques (e.g., thermal and non-thermal treatments, freezing, or synthetic chemicals). Changes in food quality induced by the application of natural preservatives have been commonly evaluated using a range of traditional methods, including microbiology, sensory, and physicochemical measurements. Several spectroscopic techniques have been proposed as promising alternatives to the traditional time-consuming and destructive methods. This review will provide an overview of recent studies and highlight the potential of spectroscopic techniques to evaluate quality changes in food products following the application of natural preservatives.

## 1. Introduction

Most food products are naturally perishable and require the application of one or more preservation and/or processing techniques to maintain their quality and extend their shelf-life. Microbial growth, chemical, and enzymatic modifications are the main alteration mechanisms, leading to the modification of the sensory and nutritional quality of most foods [1,2,3]. Some estimates indicated that about 25–30% of food is lost, due to microbial spoilage [4,5]. Alterations in nutritional and sensory properties of food can be prevented or delayed by the application of many reliable, well-established techniques, including among others low temperature-based methods (e.g., chilling, and freezing), thermal and non-thermal treatments, and traditional further processing processes (e.g., salting, smoking, and drying) [5,6,7,8]. Chemical and synthetic preservatives, such as sodium benzoate, sodium nitrite, and sulfur dioxide, have also been used (until recently) to enhance the antioxidant and antimicrobial stability of foods. However, these compounds have raised concerns about their possible implications in health problems [1,4,9,10,11]. However, the consumer’s trust in natural preservatives is strengthening. The trend towards ‘natural foods’ has also been strengthened by the ongoing COVID-19 pandemic, which has made consumers more conscious and concerned about food safety than before this crisis. Even before this pandemic, new consumer trends, e.g., low salt in muscle foods and lightly processed, sustainable, and natural products, had been documented [12,13,14,15].

A wide range of natural preservatives derived from plants (e.g., essential oils and plant extracts), animals (e.g., chitosan and lysozyme), or microorganisms (e.g., bacteriocin and organic acids) have been applied to many food products [10,16,17,18]. Known mainly for their antimicrobial and antioxidant properties, these substances are part of an increasing trend to be used as natural preservatives for several food categories, especially for those that are known to be highly perishable, e.g., muscle food products [12,16,19]. Essential oils (EO) are well-known for their antioxidant and antimicrobial properties [2,20,21,22,23], while animal-derived compounds, such as chitosan, are often applied in combination with other preservatives that can be used to preserve quality and enhance the quality parameters of treated products [17,24,25,26]. Development of edible coatings and films enriched with EO or other additives and the use of active packaging are also among the strategies used for food preservation [21,27,28,29,30,31]. Bacteriocins and other microbial-derived substances (e.g., organic acids), and bioactive compounds extracted from diverse animal and plant sources are considered as promising antimicrobials [1,19,32].

**Figure 1:**
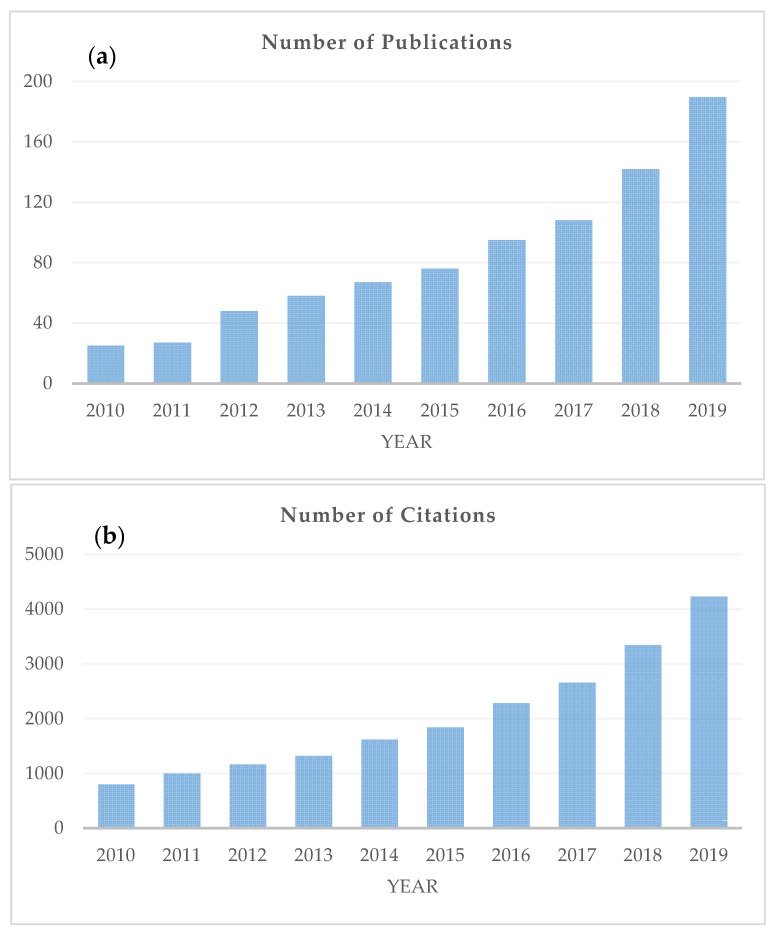
The number of publications (**a**) and citations (**b**) related to the application of spectroscopic techniques in the context of natural preservatives. Information obtained from the database Scopus (Search criteria: Article title, Abstract, Keywords: natural preservatives, OR essential oils, OR natural compounds, AND Article title, Abstract, Keywords: food, AND Article title, Abstract, Keywords: spectroscopy. The data were obtained on 3 July 2020.

Quality changes in foods, caused by the application of the aforementioned natural preservatives, have been traditionally evaluated by sensory and microbiological analysis, as well as a variety of physicochemical measurements. Although most of these methods give reliable results, they are mostly destructive and time-consuming, and thus, cannot be applied at an industrial scale for online assessment. It is, therefore, of importance to develop rapid and non-destructive techniques that can be used as alternatives to these methods. Spectroscopic techniques, e.g., Fourier-transform infrared spectroscopy (i.e., mid-infrared; MIR and near-infrared; NIR) [33,34,35], Raman spectroscopy [36,37], proton nuclear magnetic resonance (^1^H-NMR) [38,39] and fluorescence spectroscopy [40,41,42] can have an important role. Scopus (Elsevier) was searched for studies published over the past 10 years (i.e., 2010 onwards) using keywords (i.e., spectroscopy, EO, natural preservatives, food) that were relevant to this literature review (Figure 1). As can be seen from this figure, the number of Scopus-indexed articles has increased significantly during the last decade, going from 25 publications in 2010 to 191 in 2019 (Figure 1a), while the number of citations to these papers has risen from 798 in 2010 to 4233 in 2019 (Figure 1b).

This review paper will cover both the recent advances and applications related to plant-, animal- and microbial-derived preservative compounds, and the analytical methods used to assess the quality changes in food following the application of these natural preservatives. The main focus will be on antioxidant- and antimicrobial-related changes in treated foods as assessed using spectroscopic techniques.

## 2. Natural Food Preservation Technologies

Different approaches have been considered for the development of natural preservatives, which can be classified depending on their origin: Derived from plants (e.g., essential oils and plant extracts), animals (e.g., chitosan and lysozyme) or microorganisms (e.g., bacteriocins) [10,16,17,18]. However, this review has focused on EO and plant extracts (PE), target compounds for edible films and coatings, microbial-derived compounds, and other natural compounds.

### 2.1. Essential Oils and Plant Extracts

PE and EO are both natural products derived from plants with distinctive characteristics. All plants produce a variety of primary metabolites (e.g., proteins, fatty acids, and carbohydrates) and several secondary metabolites such as polyphenols, alkaloids, tannins, terpenoids, and flavonoids [43,44]. PE consists of mixtures with high concentrations of these compounds that vary depending on the plant, the extraction method, and the solvent used, usually being aqueous solutions [43,45]. On the other hand, EO are complex mixtures of natural, volatile, and aromatic compounds extracted from plants with variable compositions [23]. They are usually liquid and colorless at room temperature, and they are rich in volatile and low molecular weight secondary metabolites with their main constituents being terpenes and terpenoids, phenylpropanoids, and sulfur, and nitrogen compounds [23,46,47]. ISO 9235.2 defines EO as “products obtained from vegetable raw material (either by distillation with water or steam) from the epicarp of *Citrus* fruits by a mechanical process or by dry distillation without the addition of any water or steam”. It also allows EO to undergo physical treatments whenever the treatments do not entail significant changes in their composition [46,48]. However, even though EO have been traditionally obtained using these methods and the process has been extended to non-citrus plants, other extraction techniques such as ultrasound, microwave, or supercritical fluid extraction have been used [43,46]. Given their composition, both PE and EO have shown different biological activities such as antioxidant, antimicrobial, anti-inflammatory, or immunomodulatory [23,47,49,50]. Nevertheless, they are mainly known for their antimicrobial and antioxidant properties, both properties being applicable for food preservation [2,19]. Table 1 shows several examples of studies where EO and PE obtained from different plants were added to food matrixes along with their main beneficial properties.

Their mechanisms of action are not completely understood, but some aspects are generally agreed upon. Antimicrobial activity is usually related to the hydrophobic/lipophilic character of EO, which allow them to cross membranes and to make their different layers permeable [23]. Moreover, both PE and EO usually have high amounts of polyphenols, which have been studied for their ability to modify cell membrane permeability and to inhibit certain enzymes, among other mechanisms [50,67,68]. Antioxidant activity is usually correlated with the phenolic compounds, i.e., being able to prevent the formation of free radicals, the propagation of ROS (reactive oxygen species) and to chelate pro-oxidants [69], and thus, stopping or delaying oxidation and thereby extending the food products’ shelf-life [2]. As PE and EO can be destabilized, due to different factors (e.g., light, temperature, oxygen, and pH) when incorporated into food products, several encapsulation systems have been studied to assure and enhance the properties of these compounds [46], as well as the inclusion of these extracts in active packaging systems [70].

### 2.2. Edible Films and Coatings

**Figure 2:**
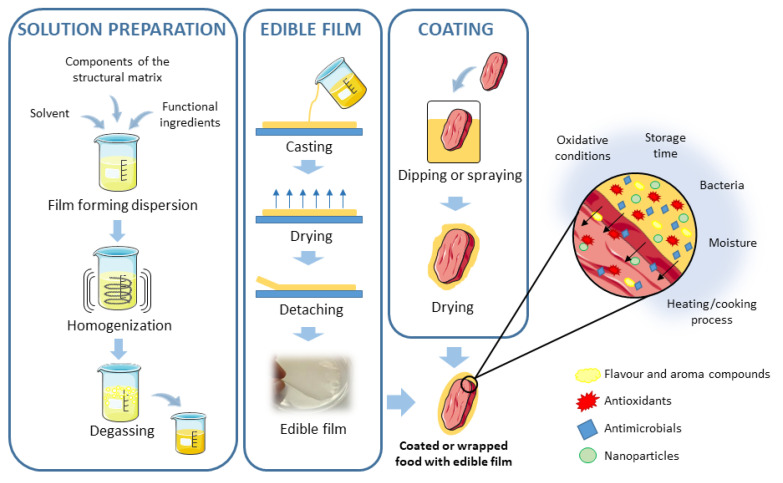
Scheme for the preparation of edible films and coatings and the different bioactive compounds that can be incorporated into them. Adapted from References [31,73].

Edible films and coatings are considered as primary packaging systems that have been proposed as alternative methods for food preservation [71]. Consumers seem to be willing to accept some products that use edible, biodegradable, and eco-friendly packaging materials [72]. Among them, polysaccharides, such as chitosan, pectin, or alginate have been used [71]. Moreover, these compounds are often applied in combination or enriched with other preservatives (such as EO and PE) to enhance the quality of food products [17,25,26]. Both techniques usually appear together, but they are different in their application process: Edible films are synthesized separately and then applied to food as a covering, whereas coatings are formed directly over the food surface by dipping the product into the coating solution [31,73]. Edible films and coatings should fulfill certain requirements that will depend on the characteristics of the food to be protected, such as low water vapor permeability or low oxygen permeability, but in general, they should: (1) Have a high water barrier, (2) control or retard gas exchange, and moisture intake, (3) confer microbial, biochemical and physicochemical stability, and (4) be economical, safe, and effectively integrated with the food supply chain [71,74,75]. Natural edible films or coatings can incorporate aroma, flavor compounds, or nutritional substances, as well as encapsulated antioxidant and antimicrobial agents, or even nanoparticles [31,71]. Figure 2 shows a scheme for how edible films and coatings are formed in different ways, and the different bioactive compounds that can be incorporated into them. Thus, even though there is no expectation that traditional packaging materials will be abandoned, the edible films and coatings are a suitable alternative for enhanced or extended shelf-life and stability of food products and the improvement of the postharvest physiological properties of some products, preventing surface contamination and possibly improving food packaging efficiency [72,73].

Polysaccharides used in these applications can be classified depending on their origin: Animal-derived compounds (e.g., chitin and chitosan), plant-derived (e.g., cellulose, starch, pectin, and gum Arabic), seaweed-derived (e.g., agar, alginate, and carrageenan), and microbial-derived (e.g., pullulan, gellan, and xanthan gum). Lipid compounds, such as oil, resins, or waxes, have also been used, and protein-based films (e.g., gelatin, collagen, milk, soy, and whey proteins) are also being studied [71]. All these compounds are supposed to be non-toxic and used as the inner layers that are directly in touch with the food products [75,76]. Table 2 lists some examples of applications of edible films and coatings based on different edible materials. Current studies have also been focused on composite films and coatings based on a multicomponent approach to take advantage of the best properties of each component, avoiding their individual drawbacks and enhancing the properties of the food [76]. All of these compounds have potential as natural preservatives and as molecules’ carriers for the enhancement of food quality.

### 2.3. Microbial-Derived Compounds

**Figure 3:**
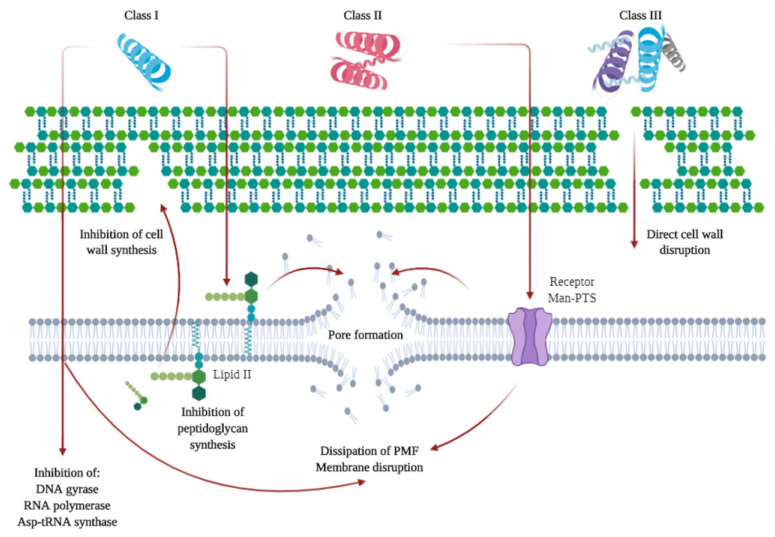
Summary of the mechanisms of action of class I, II, and III bacteriocins. Class I can directly penetrate cell membranes and affect cell integrity using different inhibition pathways. It can bind to lipid II, causing the inhibition of peptidoglycan synthesis and thus, disrupting cell wall formation or inducing pore formation that leads to cell death. In the case of Class II, they can penetrate cell walls and bind to mannose phosphotransferase (Man-PTS) receptors initiating a process of pore formation, as well as penetrate cell membranes and affect cell content by disruption of the proton motive force, among other activities. Class III can act directly on cell walls causing their disruption. Based on References [113,127,128].

As previously mentioned, some microbial-derived compounds, namely, polysaccharides, such as pullulan or xanthan gum, have been used in edible film and coating formulations. Others include bacteriocins. Bacteriocins are low molecular weight ribosomally synthesized peptides or complex proteins produced by several Gram-positive, Gram-negative, and archaea bacteria that are able to inhibit or kill other microorganisms [113,114,115]. These compounds are non-toxic and physically stable, thus they have a high potential as food preservatives for their antimicrobial properties [113]. Several bacteriocins have been isolated, especially from the lactic acid bacteria (LAB) genera, as they are GRAS (generally recognized as safe by the US Food and Drug Administration (FDA)) organisms that can be easily incorporated into food [115]. Bacteriocins can be divided into three or four different classes (depending on the authors) according to different characteristics, such as genetic profile, biochemical characteristics, the content of sulfide bonds, molecular weight, heat and enzyme stability, post-translational modifications, and antimicrobial action [113]. In general, Class I or lantibiotics are small peptides (19–38 amino acid residues) with lanthionine and methyllanthionine in their primary structure, thermostable and post-translationally modified [113,114]. The main representative of this class in nisin, which is also the only commercially produced and approved by multiple regulatory agencies for use with muscle foods for preservation purposes, among others [115,116]. Class II are heat-stable peptides, also small with an amphiphilic helical structure. Class III encompasses larger bacteriocins that are heat-labile and non-lytic with complex activity and protein structure. And class IV are more complex bacteriocins containing lipid or carbohydrate moieties and are sensitive to glycolytic or lipolytic enzymes [113,114]. Bacteriocins, after synthesis and transport, mainly act by disrupting cell walls or inhibiting the synthesis of targeted molecules. A summary of these mechanisms is shown in Figure 3. Bacteriocins can be used as isolated pure preparations, or as the products of bacteriocin-containing fermenters, bacteriocin-producing cultures, immobilized components of edible films or even combining them with nanoscale drug delivery systems [116,117,118]. They can be used in combination with EO or clinical antibiotics, sometimes with a synergistic effect [119,120]. The possibilities of bioengineering (i.e., directed mutagenesis, heterologous expression) of bacteriocins are being studied, including possible mutagenesis to improve their preservative effect, reduce the incidences of antibiotic resistance and enhance their role as health modulators of the human gut microbiota [115,116,121]. Some studies have shown the effect of bacteriocins with food products. Enterocin AS-48 showed a significant reduction of viable microorganisms and a decrease of biogenic amines when used during sardine storage [122]. A study showed that a pre-treatment with a nisin enriched osmotic solution combined with vacuum packaging could extend the shelf-life of tuna from 10 (untreated) to 51 days [123]. Nisin was also used on a nanoemulsion-based active coating mixed with EO and polylysine. It extended the shelf-life of meat products [124]. Other studies have investigated the addition of bacteriocins to food products with nanoparticles or as bacteriocin-producing cultures [125,126].

### 2.4. Other Natural Compounds

Some molecules are known for their strong antioxidant and antimicrobial character, such as the polyphenols or flavonoids [50,68]. However, other natural compounds have been used as preservative agents, such as organic acids, enzymes, or bioactive peptides. Some examples are discussed in this section. A study showed that the addition of organic acids, e.g., acetic acid and sodium lactate to pork sausages, avoided early rancidity, oxidation, or off-flavors without compromising sensory qualities [129]. Other authors researched the effect of adding lactic, citric, and acetic acids with NaCl to refrigerated brown crab meat and found that shelf-life could be extended up to 3 days by adding lactic acid and doubled by using acetic acid [130]. The enzyme lysozyme is used as a food preservative for its capacity to hydrolyze the bacterial cell wall, causing cell death [131]. Recent studies have evaluated its antimicrobial capacity using it in combination with catechin on gelatin-based edible films to maintain the quality of pork meat [132,133]. Another study assessed the effects of adding lysozyme combined with phytic acid on refrigerated grass carp fillets showing a reduction in the proportion of *Pseudomonas*, *Shewanella*, and *Aeromonas*, as well as a reduction in total volatile basic nitrogen and putrescine, thus extending their shelf-life for two days [131]. Other enzymes have also been tested, such as a lactoperoxidase system, which was incorporated into whey protein-based coatings and applied to refrigerated rainbow trout to provide a shelf-life extension of up to four days [134].

## 3. Traditional Methods Used to Evaluate Quality Changes

Quality changes occurring in food products, due to the addition of natural preservatives, can be evaluated by using several traditional methods. Sensory and microbiological measurements are the most often used, but a range of physical and chemical/biochemical parameters (such as oxidation products and volatile compounds) can also be applied. As muscle foods (e.g., fish, and mammalian and avian meats) are highly perishable, the following section will discuss the use of traditional techniques with this category.

### 3.1. Sensory Analysis

Both microbial activity and chemical changes cause quality degradation of muscle food products during processing and storage [135,136]. Natural preservatives can be used to improve sensory properties and to extend the shelf-life. Arulkumar et al. recently investigated the impact of the extract of betel leaf (*Piper betle*) on sardine (*Sardinella albella*) meat to prolong shelf-life during storage in ice [137]. They found that the extract treatment enhanced the sensory qualities of fish and prolonged its shelf-life by 12 days compared to the control group. In another study, Gao et al. reported that epigallocatechin gallate-gelatin biofilm treatment efficiently lessened the fishy smell of tilapia, enhanced the sensory quality, and extended the shelf-life of the fish fillets for six days at 4 °C [138].

On the other hand, when used at high concentrations, natural preservatives, such as EO, may result in undesirable sensory properties of treated seafood products [2,139] and meat products [140,141]. Actually, some EO have a strong odor and flavor, thus they may leave an undesirable after taste, which could result in a reduction in the acceptance or liking degree for meat and seafood products [142,143,144]. Cilli et al. assessed the effect of 1 and 2% grape pomace flour (GPF) addition on frozen salmon burgers in terms of oxidative stability, physicochemical, and sensory characteristics [145]. The authors observed that GPF decreased the degree of liking of appearance, color, and overall quality of salmon burgers compared to the control groups (*p* < 0.05). Moreover, using 2% GPF generally resulted in a lower flavor score, as it masked the characteristic fish flavor.

Color and texture appear to be the most relevant sensory properties affecting consumer purchasing decisions of foods [146,147]. The L* color parameter describes the ‘‘lightness” and usually lessens in meat and meat products during extended refrigerated storage because of oxidation [148]. Chuesiang et al. investigated the effect of low (LC) and high levels (HC) of cinnamon oil nanoemulsion (CN) and bulk cinnamon oil (BO) on the quality of Asian seabass (*Lates calcarifer*) fillets [149]. Throughout storage, while L* values of the treated samples stayed constant, a* (the green–red component) and b* (the blue-yellow component) values were significantly decreased and increased, respectively, particularly for HC-CN, and HC-BO treated fillets. In another study, He and Xiao reported that the tangerine peel (TP) EO glazing layer treatment significantly (*p* < 0.05) influenced the hardness, springiness, and cohesiveness of bream (*Megalobrama amblycephala*) samples during storage [150].

### 3.2. Microbiological Evaluation

Natural preservatives, especially EO, have antimicrobial properties that have been widely exploited in the seafood processing industry [2,151]. For example, antimicrobial and antioxidant properties of EO (oregano, thyme, and star anise) on microbial load and quality of grass carp fillets were studied by Reference [152]. EO treatments were observed to be effective in preventing microbial growth and retarding lipid oxidation, and therefore, prolonging the shelf-life of grass carp fillets (six days for the control and eight days for EO groups). Nano-emulsions based on EO have an antimicrobial effect against most Gram-positive and Gram-negative bacteria. It was reported that nanoemulsions based on EO of rosemary, laurel, thyme, and sage improved the organoleptic quality of rainbow trout. Nano-emulsions based on EO inhibited the growth rate of mesophilic anaerobic bacteria, psychrotrophic bacteria, and Enterobacteriaceae on rainbow trout fillets [153]. Similar results were reported for cinnamon oil nanoemulsions for Asian seabass (*Lates calcarifer*) fillets [149].

Some studies investigated the possibility of adding plant extracts directly to the ice used for the preservation of fish. For example, the impact of the rosemary extract (0.05 and 0.1%) with icing on the quality of sardines was evaluated using chemical, sensory, and microbiological procedures [55]. The ice containing rosemary extract reduced the growth of bacteria, thus maintaining the quality and safety of the sardines. In a similar study, the addition of an extract of thyme (0.04% w/v), oregano (0.03% w/v), and clove (0.02% w/v) in ice used for the storage of anchovies resulted in a longer shelf-life [154]. He et al. studied super chilling storage-ice glazing (SS-IG), applying 0.1, 0.2, and 0.3% v/v of clove EO mixtures on sea bass [146]. Results indicated that the mixture was efficient in the prevention of microbial and chemical spoilage of fish samples. Yavuzer et al. [155] also investigated the effect of icing with peel extracts of potato, sweet potato, sugar beet, and red beet on the sensory, chemical, and microbiological attributes of rainbow trout fillets. One of the main conclusions was that these waste products could be used as antimicrobial agents. Li et al. suggested that the use of grape seed extracts (GSE) for cultured snakehead fillets prevented the growth of *Aeromonas* [156]. The use of laurel and myrtle extracts significantly reduced bacterial growth in European eel fillets [157]. Lahreche et al. [158] also reported low bacterial growth when both oregano extract and vacuum packaging were applied to frigate tuna muscles. And carvacrol was reported to extend the shelf-life of sea bass fillets, due to its inhibitory effects on *Pseudomonas*, H_2_S-producing bacteria, *psychrophilic* bacteria, lactic acid bacteria (LAB), and yeast and molds [159].

Chitosan coatings, along with EO, have been used in food products, due to their properties being biocompatible, transparent, and colorless with good antimicrobial activity [160,161]. Valencia Junca et al. used chitosan beads incorporated with EO of *Thymus capitatus* with red tilapia fillets [161]. Shelf-life of treated groups increased, due to the inhibition of growth of microorganisms, including total coliforms, *S. aureus, Vibrio parahaemolyticus*, and *Salmonella enterica* sub enterica. The efficiency of pectin coatings supplemented with clove (C) EO, as edible coatings were used to maintain the quality of bream fillets during 15 days of chilling [161]. Pectin coating with CEO was efficient in preventing bacterial growth, particularly in Gram-negative bacteria, whereas the growth of LAB stayed steady for almost the entire storage period.

### 3.3. Physical and Chemical/Biochemical Parameters

Consumers are more aware of health concerns and may no longer approve chemical preservatives, due to possible toxicological effects. Therefore, natural extracts and products prepared in an environmentally friendly way have been applied to, among others, prevent the formation of BA in fish and seafood products. Noori et al. reported a decrease in the production of BA in common carp treated with both lactic acid and ajwain (*Carum copticum*) [162]. Histamine was not detected at the initial stages of common carp stored at 4 °C, in agreement with the results of Hosseini et al. [163]. Özogul et al. investigated BA formation of vacuum-packed European anchovy along with 1% lemon balm (LB) and lavender (LD) extracts [151]. Both LB and LD extracts decreased most BA content, especially lessening histamine 3-fold compared to the control group.

Lipid oxidation and protein degradation are caused by bacteria and endogenous enzymes [152]. Many plant extracts and their EO have been observed to have positive influences on retarding lipid and protein oxidation and prolonging the shelf-life of various fish and seafood products with different storage conditions. The microbiological features and quality attributes of turbot fillets treated with several concentrations (1, 4, and 8 μL/L) of EO of clove, cumin, and spearmint for 20 days at 2 ± 1 °C were studied [164]. All EO inhibited the oxidation of turbot fillets, and the most efficient EO was spearmint oil. Spearmint oil at 4 μL/L preserved the color and texture, delayed the protein and lipid oxidation, and decreased the bacterial counts.

Both peroxide value (PV) and thiobarbituric acid (TBA) are important parameters to measure the level of lipid oxidation during food storage [27]. The use of rosemary extract, a good antioxidant source, in combination with nisin, was investigated to prolong the shelf-life of pompano (*Trachinotus ovatus*) fillets during refrigerated storage [165]. Lipid oxidation was greatly reduced using rosemary extract reflected as decreased PV and TBA values compared to the control and nisin group. Rosemary extract inhibited lipid oxidation by scavenging free radicals to stop the free radical chains. Similar antioxidant properties were observed with grape pomace flour [145], chlorogenic acid [166], and gelatin-polycaprolactone composite films combined with lysozyme and pomegranate PE [136].

Another parameter that has been used as a quality change indicator is total volatile basic nitrogen (TVB-N), whose increase during storage results from microbial activity. The lowest TVB-N increase for mackerel was obtain using a composite film combined with pomegranate PE-lysozyme [136]. This was probably due to the bioactive polyphenols in pomegranate PE and antibacterial properties of lysozyme, which prevented the growth of microorganisms responsible for the oxidative deamination of non-protein nitrogen compounds. The impact of sodium alginate (SA) coating, along with tea phenolic compounds (TP) on the quality of fresh Japanese sea bass fillets was evaluated [167]. The results showed, among others, that the SA-TC treatments reduced the TVB-N. The lowest TVB-N content was also obtained from refrigerated Chinese shrimp treated with chitosan coatings combined with carrageenan and ε-polylysine, which inhibited microbial growth and delayed the microbial oxidative deamination of nitrogen-containing compounds [168].

The ATP catabolism pathway in fish has been studied, and the breakdown is as follows: ATP, ADP, AMP, IMP, HxR, and Hx, which then goes on to other compounds. The amount of each of these compounds can be used to determine the K-value in many fish species. Ocaño-Higuera et al. reported that K-value shows the change in freshness, especially at the initial stage of storage [169]. *Allium sativum* could prevent the degradation of ATP and retain the freshness of large yellow croaker [170]. Huang et al. reported that cinnamon bark EO can efficiently prevent the degradation of ATP and maintain the high quality of grass carp fillet [171]. Chitosan at 2% was found to be efficient in delaying the degradation of ATP, thus lower the K-value, extending the shelf-life of fish samples in frozen storage [169].

## 4. Spectroscopic Monitoring Techniques

### 4.1. Overview of Spectroscopic Techniques

Spectroscopic analytical methods are commonly used in food quality assessment, due to their non-destructive nature and versatility of applications [172,173]. The main spectroscopic methods are based on two fundamental concepts, i.e., vibrational and spin-based spectroscopy. The most commonly applied spectroscopic methods in food science are described in this section. The main advantages and limitations of each spectroscopic technique are summarized in Table 3.

The vibrational spectroscopy methods can best be described using the quantum effect of electromagnetic energy excitation and emission, as shown in a Jablonski diagram [174]. Absorption spectroscopy methods ranging from the ultraviolet (UV) region of the electromagnetic spectra to the infrared (IR) region correspond to the excitation of a molecule through the absorption of light from the ground state (S_0_) to an excited state (S_1_). This requires a specific quantity of electromagnetic energy (ΔE), which is characteristic of the chemical composition and environment of the molecule. On the other hand, fluorescence and phosphorescence spectra arise, due to emission from an excited quantum state back to the ground state, generally at a higher wavelength with less electromagnetic energy [175,176].

The mid-infrared (MIR) wavelength domain (wave numbers from 4000–400 cm^−1^) gives rise to fundamental tones of the natural vibrations of bending, stretching, twisting, etc. of chemical bonds. Vibrations of common functional groups found in food products, such as -OH, -NH_2_, -CH_3_, C=O, and C_6_H_5_- absorb and generate strong and narrow signals in the MIR [35,174,177]. These absorption bands are dependent on the effects of hydrogen bonding, water content, particle size, temperature, etc. The positions of the fundamental absorption bands are well documented [178,179], making IR spectroscopy an effective tool in the analysis of proteins, carbohydrates, etc. Fourier transform (FT) IR has been used for various applications, including monitoring of quality changes in foods as a result of the addition of natural preservatives [34,180,181,182].

The near (N) IR (wave numbers from 12500–4000 cm^−1^) domain mainly give rise to overtones and combination bands of the fundamental tones seen in the MIR. Vibrational overtones of common biochemical bonds, such as C-H, O-H, N-H, S-H, and C=O are detected in the NIR, making NIR spectroscopy useful for both qualitative and quantitative analysis of water, lipids, proteins, and carbohydrates [174]. However, the peaks in the NIR are often broad and overlapping, often making peak assessment and interpretation of the spectra difficult, unless coupled with multivariate chemometric analysis. NIR spectroscopy has been shown to be an effective and fast method for online assessment of various food quality parameters.

Raman scattering is based on inelastic light scattering and changes in molecular polarizability, allowing even normal IR-inactive vibrations to produce signals, due to their fundamental molecular vibrations [183]. Surface-enhanced techniques are the most sensitive way to use Raman scattering, allowing the analysis of complex functional and structural characteristics of single biomolecules [184].

UV-Vis spectroscopy is mostly used for destructive studies, where chemical reactions leading to the development of “color” are used. For example, the analysis of oil degeneration by measuring anisidine and peroxide values (AV and PV) of oils, which form complexes that give rise to peaks in the UV spectral range (50,000–25,000 cm^−1^ or 200 to 400 nm). However, the visual spectrum (Vis, 25,000–12,500 cm^−1^, or 400 to 800 nm) allows analysis of the color of samples and any changes [174,185]. This makes the visual spectrum important, especially in the assessment of the performance of natural preservatives. Fluorescence spectroscopy, as mostly used, is based on the emission of light from a fluorophore, a fluorescent molecule, which has been excited by either UV or visible light. This gives rise to both an excitation and an emission spectrum that can be used for analysis. Fluorescence is seldom used alone for food analysis, but rather in connection with molecular separation techniques, such as liquid chromatography, e.g., high-performance liquid chromatography (HPLC), which allows the molecules involved in fluorescence to be identified. Such combinations of techniques detect low concentrations of pathogenic microbes, toxins, antibiotics, or food additives, and is a powerful method to detect the causes of food poisoning [174,186]. Fluorescence spectroscopy may also be used for the analysis of structural changes of proteins, characterization of carbohydrates and lipid classes in oils, among many other applications [174,175,187].

Nuclear magnetic resonance (NMR) is based on the spin of nuclei with a magnetic momentum, such as ^1^H, ^13^C, ^23^Na, and ^31^P that are excited between energy states, created when the molecule is placed in an external magnetic field. Electromagnetic pulse trains are used to excite and change the spin direction of the nuclei. After the pulses have been turned off, the nuclei return to their fundamental spin state. The time it takes the nuclei to return to the fundamental state is characteristic for the molecule and their environment [188]. NMR analytical methods are often classified according to the external magnetic field strength applied. Low field NMR (10–50 MHz) is used for several applications of food quality assessment and has been shown to be able to effectively measure several water and lipid characteristics of food, changes in muscle structure and functionality, characteristics of emulsions, etc. [189]. High field NMR (>300 MHz) has been used for a detailed analysis of the chemical structures in organic chemistry and pharmaceutical applications. In food science, high field NMR has been especially useful in structural analysis of complex molecules, such as proteins, carbohydrates, and lipids [190].

All the above-mentioned methods can be integrated with image analysis, resulting in methods, such as hyperspectral and magnetic resonance imaging (MRI). These methods provide pixelwise spectral and image data—which is particularly useful when samples are heterogeneous and chemical components, are not evenly distributed throughout the sample [178,191]. The development of more handheld and mobile instruments, which allow faster quality analysis where traditional laboratory instruments did not have easy access, are now available.

### 4.2. Examples of Applications of Spectroscopic Techniques

#### 4.2.1. UV-Vis and Fluorescence Spectroscopy

The UV-Vis spectroscopy is based on the measurement of the absorbance of the sample in the region of the UV (250–400 nm) or visible (400–780 nm) spectrum, and reflects the energy absorbed for electronic transitions [7]. To evaluate the total antioxidant capacity (TAC), which has several health benefits, the spectroscopic methods can be used, and the evaluation is based on the generation of radical species and their subsequent disappearance when the antioxidant compound is added [192]. For example, an in vitro antioxidant test is the Trolox equivalent antioxidant capacity (TEAC) assay. This method measures the decrease in the color of the ABTS^•+^ reagent when returning to its initial form as ABTS, due to the antioxidant effect of the sample, and is normally measured at 734 nm [193]. Another widely used test is the DPPH test, which is based on the same principle as TEAC. DPPH is a dark purple reagent that loses its color when the antioxidant sample is added, and is normally measured at 515 nm [193]. Several studies can be found in which these methods are used to assess the quality of foods enriched with natural preservatives. Brodowska et al. evaluated the preservation of ground pork patties with sour cherry extract for eight days by measuring the antioxidant capacity using TEAC [194]. In another study, Pasukamonset et al. also used the TEAC assay to evaluate the oxidative stability of cooked pork patties with *Clitoria ternatea* extract [195]. Lorenzo et al. [196] used the TEAC assay to evaluate the influence of pitanga leaf extracts on the TAC of pork burgers during storage. On the other hand, Al-Juhaimi et al. used the DPPH method to evaluate the antioxidant potential of *Acacia nilotica* fruit flesh extracts in beef burgers [197].

Spectrophotometric methods are also useful to determine lipid oxidation of food products. Lipid oxidation promotes the degradation of polyunsaturated fatty acids (PUFA) and the production of carbonyls and hydrocarbon compounds as decomposition products. Lipid oxidation is the main factor that influences the quality of meat and its derivatives, since it entails changes in quality parameters, such as aroma, color, flavor, and/or nutritional value [198,199]. The thiobarbituric acid reactive substances (TBARS) assay is used to evaluate lipid oxidation. This test consists of the measurement at 532 nm of the colored complex formed between malondialdehyde (MDA) and 2-thiobarbituric acid (TBA). One application has been to test the addition of natural preservatives with meat products, which have been investigated in a large number of studies. Some of these studies have already been discussed [27,136,145,164,165,166]. Other meat quality parameters, such as conjugated dienes (measured at 233 nm), protein carbonyl groups (measured at 370 nm), protein thiol groups (measured at 410 nm), or metmyoglobin (measured at 525, 572, and 730 nm), all of which reflect quality and acceptability can be measured by UV-Vis spectroscopy [195,200].

Two studies were conducted to study the potential of using fluorescence spectroscopy to investigate the impact of glazing with rosemary (*Rosmarinus officinalis*) extract [201] and glazing with polyacrylate and D-sodium erythorbate [202] on the preservation of mud shrimp (*Solenocera melantho*) and the quality changes of squid during frozen storage, respectively. In these two studies, the intrinsic fluorescence of tryptophan was used as an indicator of conformational changes of myofibrillar proteins. Results showed a smaller decrease in fluorescence intensity of glazed samples compared to the controls, indicating a protective impact of the glazing treatment against protein degradation and denaturation. Cropotova et al. used fluorescence spectroscopy successfully to monitor changes in quality (especially oxidation measured using TBARS) of Atlantic mackerel (*Scomber scombrus*) subjected to different natural antioxidants (rosemary extract and rosemary extract with ascorbyl palmitate) and sous-vide cooking treatments [41]. In another study, the same fish species treated with rosemary and basil EO was also analyzed using fluorescence spectroscopy and several traditional quality changes indicators [40]. Although neither sensory nor microbiological measurements were done, the results of the other traditional techniques seemed to indicate an increase of 2 to 5 days in the shelf-life of the treated samples. Moreover, high correlations were observed between fluorescence data and traditional measurements, suggesting that fluorescence spectroscopy could be used as an alternative to traditional measurements.

#### 4.2.2. Vibrational Spectroscopy

**Figure 4:**
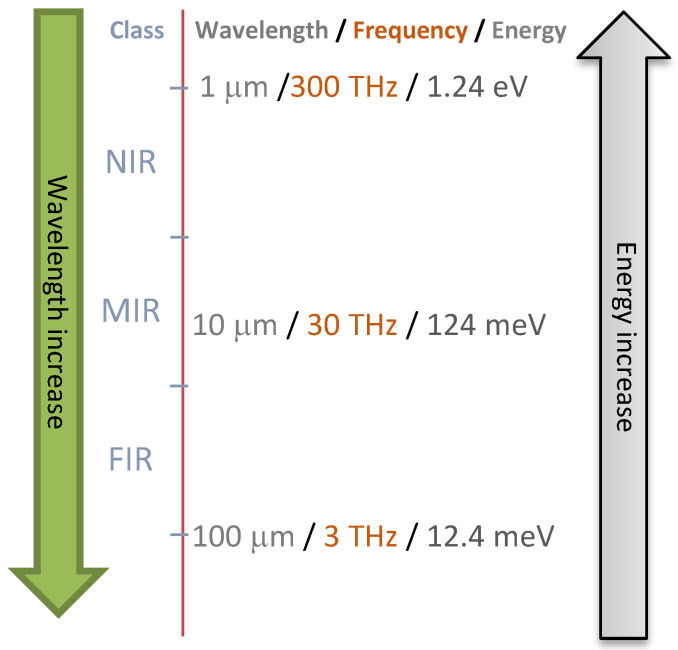
Vibrational spectroscopies are commonly used in the assessment of food quality and safety.

Vibrational spectroscopy over a range of wavelengths (Figure 4) can be used. Rapid monitoring of the spoilage of minced beef stored with conventionally and active packaging conditions using FT-IR with chemometrics was investigated by Ammor et al. [182]. Their results showed the ability of FT-IR with chemometrics to accurately predict the packaging type. They also developed a model that could accurately discriminate minced beef samples according to the type of packaging. This could be an economical, rapid and non-invasive tool for the monitoring of minced beef spoilage using the measurement of biochemical changes rather than enumerating bacteria [182]. Computational methods using classification or regression models from spectral or imaging data can be used for model training and validation [203]. The results showed that FT-IR spectral data could be potentially used to monitor spoilage as it can be correlated with microbial counts and/or organoleptic characteristics. Pavli et al. showed that high-pressure processing and the addition of oregano EO in sodium alginate edible films with meat products can eliminate the growth and even the presence of *Listeria monocytogenes* [181]. The use of hyperspectral imaging technique for the non-destructive prediction of pork meat quality and safety was shown by Tao and Peng using a real-time detection system for evaluating pork meat tenderness and *E. coli* contamination [204].

#### 4.2.3. Raman Spectroscopy

Raman spectroscopy (RS) requires minimal or no sample preparation and can be used for online quality control. Although there is low sensitivity, due to RS intensity, of about 10^−6^ fold that of the excitation laser, which limits its practical application. However, many enhanced techniques have been developed, such as resonance Raman spectroscopy (RRS), coherent anti-Stokes Raman spectroscopy (CARS), stimulated Raman spectroscopy (SRS), surface-enhanced Raman spectroscopy (SERS), and tip-enhanced Raman spectroscopy (TERS) that improve the intensity of RS. Among these technologies, SERS is the most used and effective [205]. The FT-RS has been used to investigate the role of antioxidants and cryoprotectants in minimizing protein denaturation in frozen cod (*Gadus morhua*) fillets treated with either antioxidants (vitamin C or vitamin C with vitamin E), antioxidants (vitamins C + E) with citrate, cryoprotectants or their mixtures. Raman structure analysis showed differences in the frequency, intensity, and the shapes of the peaks, indicating that the antioxidants minimized the denaturation of proteins caused mainly by ice crystal formation and/or the resultant solute concentration, and also lipid oxidation products. Furthermore, the denaturation of proteins involved an increase in hydrophobic groups and exposure of other polar groups, due to the unfolding of the molecules, accompanied by changes in the secondary structure [206].

The antioxidant effect of an innovative active plastic film containing olive leaf extracts on fresh pork meat was evaluated using Raman spectroscopy to determine the efficiency of the active materials on the inhibition of oxidation and the extension of the shelf-life of fresh meat, as well as showing the feasibility of Raman spectroscopy for monitoring of meat oxidation [37]. Raman spectroscopy showed that the antioxidant effect increased when the concentration of natural extract in the multilayer film increased. The authors also referred to the Raman bands that can be used for the determination of total unsaturation. In another study, it was reported that clove and cinnamon EO prevented lipid and protein oxidation and extended the shelf-life of ready to cook (RTC) pork chops during chilled storage. Raman spectroscopy provided data correlating with TBARS values and allowing a better understanding of the structural changes in RTC pork chops during refrigerated storage [207]. The effect of adding several antimicrobial and antioxidant additives (sodium citrate tribasic, E331, ascorbic acid, E300, and sodium ascorbate, E301 on meat drip from defrosted yellowfin tuna fish loins was studied by Howes et al. [208]. Different bands of Raman spectra, especially those located at 570 and 578 cm^−1^ allowed to gain insight into the nature of the complex formed in the presence of these additives.

#### 4.2.4. NMR and EPR Spectroscopy

The applications of magnetic resonance techniques, particularly NMR and electron paramagnetic resonance (EPR) has not yet been recognized as an official methodology of food analysis despite numerous potential applications. Nonetheless, they are contributing to food science and complementing other conventional methodologies [209,210]. The main reasons for the increase of EPR/NMR-related food applications are the development of more powerful methods, as well as technological breakthroughs, benchtop instruments, and user-friendly software. The most important difference between NMR and EPR is that the NMR is concerned with the changes in spin in the nucleus while EPR describes changes in the electron spin. EPR, also called electron spin resonance (ESR), is a lesser-known magnetic resonance technique, which utilizes microwave radiation to probe species with unpaired electrons, such as radicals, radical cations, and triplets in the presence of an externally applied static magnetic field. Although limited to samples with unpaired electron spins, EPR spectroscopy has a broad range of versatile applications. Both techniques of magnetic resonance could provide information about the structural, compositional, and functional aspects of molecules. High-resolution NMR spectroscopy, applied to the liquid, semi-solid, or solid food samples can be used to determine chemical composition, including authentication and classification, quality control, sensory evaluation, understanding molecular mechanisms and interactions of food components, as well as the reactions of antifungal and antibacterial effects [210,211,212,213]. Low field (LF) NMR relaxometry and imaging applied to food samples gave information regarding their morphology and texture [214]. Magnetic resonance imaging (MRI) is a technique used to monitor the spatial, internal morphology, and molecular distribution of water in marine fish.

NMR studies of natural preservatives for meat and fish products have been used to measure changes in quality. By monitoring the NMR transverse relaxation T_2_ of the three different types of water Liu et al. found that the application of gelatin with red pitaya methanol PE (RPPE) as a coating for crayfish (*Procambarus clarkii*) during refrigerated storage can be effective in retarding quality deterioration [215]. The results showed an increase in free water and slight changes of bound and immobile water in deshelled crayfish samples with storage. There was no significant differences detected among the immobilized water for treatment with gelatin solutions containing 0.1% (w/v) ε-PL with 3% RPPE compared with other samples (0.1% (w/v) ε-PL with 1% RPPE and 0.1% (w/v) ε-PL with 2% RPPE). The authors concluded that probably due to the greater RPPE addition, the immobilized water within the myofibril movement to free water was slowed, maintaining the good quality of the crayfish muscle [215]. Li et al. applied LF-NMR as an effective way to evaluate the freshness of farmed Japanese sea bass (*Lateolabrax japonicus*) during cold storage with the application of sodium alginate (SA) and flaxseed gum (FG) active coatings containing microencapsulated eugenol emulsions for extension of shelf-life [216]. The results showed that sea bass samples treated with eugenol had a lower content of free water. The authors also monitored water migration using MRI and the proton density-weighted images of sea bass samples, and confirmed that on the 16th day the color of uncoated sea bass samples were darker and bluer than others because of myofibrillar degradation, whereas the brightness of eugenol treated sea bass samples were lighter compared with the uncoated ones. SA-FG coatings indicated that 0.15% eugenol addition combined with cold (4 °C) could be suitable for retaining the freshness and extended the shelf-life of the sea bass samples. Similar results were reported after glazing with sodium polyacrylate (SP) and D-sodium erythorbate (DSE) on the quality changes of squid during the frozen storage of squids. NMR results showed that compared with the unglazed groups, the application of glazing had a beneficial effect on moisture loss and prolonged shelf-life [202]. Glazing tuna with rosmarinic acid, a bamboo leaf antioxidant, and sodium lactate that was stored at −18 °C for 180 days showed that bamboo leaf had the best water retention and could effectively reduce water losses. After the application of rosmarinic acid and sodium lactate, the data showed an uneven distribution of moisture [39]. Zhang et al. indicated that the surimi processing by-products (SPB) hydrolysates could be used as natural antioxidants-cryoprotectants, as well as gel texture enhancers in freshwater fish surimi [187]. These effects of SPB hydrolysates on silver carp (*Hypophthalmichthys molitrix*) surimi were monitored, and it was shown that SPB hydrolysates effectively enhanced the interaction of water molecules and myosin molecules within the gel network and stabilized the gel structure, which prevented the loss of water holding capacity of the gels [187]. Modified atmosphere packaging (MAP) effects with different gas ratios combined with gelatin coatings containing eugenol on Chinese sea bass were studied using high field NMR and MRI [214]. The results showed prevention of water loss and shelf-life extension. Pseudo-color images were generated from MRI depending on the level of portion density, providing visual information of the internal morphological organization and molecular distribution in the samples. The same research team obtained similar results when the effects of gelatin active coating containing eugenol/β-cyclodextrin emulsions combined with superchilling [217]. Zhu et al. [218] that the effect of nutmeg (N, *Myristica fragrans Houtt*) EO encapsulated solid liposomes (NEO-SLP) and cold nitrogen plasma treated NEO-SLP (P-NEO-SLP) were studied with pork tenderloin meat batters. The results of water holding capacity measured by NMR showed that more water was entrapped in the protein gels after P-NEO-SLP addition, due to the increased and strengthened protein-protein interaction as more proteins that were less oxidized were involved in protein entanglement [218].

EPR spectroscopy is mainly used for quantification of radical species, exploration of redox mechanisms in foods, assessment of the antioxidant and antimicrobial activity, as well as monitoring food quality through studies of protective activity, stability, and food shelf-life [209,219]. Some EPR studies have been done on natural preservatives without application in foods [220,221,222] or to follow specific food treatments, such as sterilization. Paari et al. showed using EPR radical scavenge measurements that the addition of curcumin improved the oxidative stability of irradiated goat fish (*P*arupeneus *Indicus*) [223]. Using EPR spin trapping, Bolumar et al. monitored the formation of radicals during high pressure (HP) processing of beef loins and chicken breasts [224]. The addition of natural antioxidants (rosemary extract (RE), caffeic acid, and ascorbic acid) did not provide protection against HP, while similar spin adducts for beef without antioxidant were observed. Other than iron-derived radicals, the results of this study provided evidence of the generation of protein-derived radicals during the HP-treatments. On the other hand, lipid-derived carbon-centered alkyl radical formation was studied in roasted meat using EPR. It was shown that the 0.03% addition of tea polyphenols (TPP) significantly reduced radical formation during roasting, while the 0.03% RE had no significant inhibitory effect [225]. The tendency of pork patties to form radicals was monitored using the spin-trapping technique combined with ESR at the surface and the inner parts for the different packaging systems (vacuum, RE active, and oxygen scavenger packaging) throughout storage. It was shown that RE active packaging was the most effective method to protect pork patties from the lipid oxidation induced by HP processing [226]. Niseen et al. used direct EPR measurements of low water activity products for the detection and quantification of oxidation in dehydrated chicken meat [227]. Four natural antioxidants were applied, and the results showed that RE had higher antioxidant efficiency in preventing the oxidation than coffee and tea, while grape skins had the lowest efficiency. EPR oximetry, which is based on the broadening of ESR-lines in the presence of oxygen, is a method to investigate local oxygen concentrations, oxygen diffusion products, as well as for the study of oxygen transport and the depletion of membranes and proteins. Despite its numerous possibilities for oxygen measurements, it has only been used with model food systems [228].

## 5. Final Remarks and Future Trends

Natural preservatives can be used as promising alternatives to other preservation methods. Given the consumer demand for safer and more natural foods, natural preservatives have been a research focus. The main benefits of applying natural compounds, e.g., EO, PE, and chitosan, are enhanced antimicrobial and antioxidant stabilities of the treated foods. Therefore, the use of such preservation technologies helps to maintain the quality of fresh food products and prolong their shelf-life. Nevertheless, the use of natural preservatives still has some shortcomings that prevent their broader range of industrial applications. One obstacle is the impact of some natural preservatives, such as EO or chitosan, on the organoleptic properties of treated products. Some studies have indicated that the application of the hurdle concept by combining several preservative strategies may overcome this limitation. The safety of natural preservative compounds could be an issue that needs to be addressed and require more investigations to reassure consumers. Further studies are also required to better understand this synergistic combination of different preservative approaches and how such strategies could achieve more desirable preservation. In addition, active molecules responsible for the preservative actions, the minimum quantity required to produce the intended effects, and the mechanisms of action of EO, biopreservation, and other natural preservation strategies have to be better elucidated. At the same time, the increasing consumer demand for foods of high quality and safety has led to the development of rapid, accurate, and non-destructive analytical methods that can be used in place of traditional analytical ones. Although sensory and microbiological methods have been used and considered as standard methods for the evaluation of food quality and validation of shelf-life, these methods are time-consuming and can be expensive. Other traditional methods, such as physicochemical measurements, are destructive and may require chemical treatments of samples. Therefore, several spectroscopic techniques have been tested for monitoring changes in food quality, resulting from the application of natural preservatives or other preservative strategies. The main advantage of the application of spectroscopic techniques is the non-targeted nature of the measurements, thus providing valuable information, which can be considered as a fingerprint. Most of these techniques are fast and require minimal sample preparation and/or use of chemicals, making them appropriate for the modern food industries. Among these techniques, IR spectroscopy seems to be the most used technique, while further work on other spectroscopic methods, including fluorescence. Raman and NMR is needed. Imaging, miniaturization, and handheld spectroscopic devices are examples of recent research advances. Accessible and even portable spectrometers (e.g., Raman, NIR) are already in use in some applications, while other spectroscopic instruments are still being improved. Development of software tools and packages that allow real-time analysis are among the most promising advances. These types of software have been developed in recent years and have been most useful for expanding the use of hyperspectral imaging, particularly enabling online monitoring.

Despite the desirable features of spectroscopic techniques, there still seems to be a reluctance to adapt these analytical methods industrially, due to several challenges. Many of the applications of spectroscopic techniques are only feasibility studies done at the laboratory scale or with a controlled environment. Some of these challenges are related to the techniques themselves (e.g., high cost, massive data volume), while a high heterogeneity in some food products, such as muscle foods, makes them more difficult to scan. Multiple chemometric modeling methods and variations between the reviewed studies in terms of the number of samples, validation procedures, processing conditions, among others, make it difficult to compare and interpret the results obtained from these studies. Other obstacles are more general and are related to a negative mentality toward new technologies and the lack of academic and applied education and knowledge regarding the use of these techniques. Continuous education, training, and further research could overcome some of these limitations and increase the awareness of the importance of these new technologies, opening doors for further implementation in the industrial environment. Other possibilities include more development of chemometric tools and the use of fusion data that merges the analytical information from several spectroscopic techniques. Recent studies have shown that the models built using data fusion strategies are more robust than those obtained from a single technique.

## 6. Conclusions

In summary, the application of ‘natural’ approaches to extend the shelf-life of food seems to be an interesting option and is gaining interest. The possibility of applying rapid analytical methods for monitoring quality and detecting changes in food products taking place during processing or preservation has been successfully shown. However, the path for the implementation of new technologies in industrial production systems is not straightforward. Further development and advancements of both preservation strategies and analytical methods are expected in the future, thus encouraging wider industrial applications of natural preservatives and spectroscopic techniques.

## Figures and Tables

**Table 1:** Examples of the beneficial properties of adding plant extracts (PE) and essential oils (EO) to different food products.

Plant	Product	Properties	Ref.
***Essential Oils***			
Thyme *(Thymus capitatus)*	Semi-skimmed ultra-high-temperature (UHT) Milk	Antimicrobial activity against *Staphylococcus aureus*, *Bacillus licheniformis*, and *Enterococcus hirae* Improve oxidative and fermentative stability	[51]
Clove (*Syzygium aromaticum*) *C*innamon (*Cinnamomum zeylanicum*), Myrtle (*Myrtus communis*, and Lavender (*Lavandula stoechas*)	UHT Milk	Antimicrobial activity against *Escherichia coli*	[52]
*Trachyspermum ammi* fruit	Wheat and chickpea	High antifungal activity against *Aspergillus flavus* and anti-aflatoxigenic activity against aflatoxin B_1_	[53]
*Mentha cardiac* L.	Dry fruits	High antifungal activity against *Aspergillus flavus* and anti-aflatoxigenic activity against aflatoxin B_1_	[54]
Rosemary (*Rosmarinus officinalis*) and basil (*Ocimum basilicum* L.)	Atlantic Mackerel (*Scomber scombrus*) fillets	Delay of the development of lipid oxidation and the formation of TVB-N. Extension of shelf-life of products of 2–5 days compared to the control samples	[40]
Rosemary (*Rosmarinus officinalis*), thyme (*Thymus vulgaris*), laurel (*Laurus nobilis*), and sage (*Salvia officinalis*)	Rainbow trout (*Oncorhynchus mykiss*)	Antimicrobial and antioxidant properties Enhancement of the organoleptic quality of fish	[55]
Orange (*Citrus sinensis* (L.) Osbeck)	Pink shrimp (*Parapenaeus longirostris*)	Antioxidant activity and antimicrobial activity (total viable counts, psychrotrophic bacteria, and *Enterobacteriaceae* family). Shelf-life extension of nearly 10 days	[56]
*Bunium persicum*	Iranian cheese	Antioxidant activity, antibacterial properties against *Salmonella typhimurium*, *Escherichia coli*, *Staphylococcus aureus*, and *Listeria monocytogenes*	[57]
Thyme (*Thymus vulgaris*)	Semi-solid coalho cheese	Antimicrobial activity against *Staphylococcus aureus* and *Listeria monocytogenes*	[58]
*Zataria multiflora* Boiss.	Gouda cheese	Decrease in the biogenic amines content (*Tyr* and *His*) and antimicrobial activity, especially against yeasts	[59]
Oregano	Fresh lettuce	Antimicrobial activity against *Listeria monocytogenes*, *Salmonella typhimurium* and *Escherichia coli*	[60]
***Plant Extracts***			
Guarana (*Paullinia cupana*) and pitanga (*Eugenia uniflora* L.)	Lamb burgers	Reduction of the lipid and protein oxidation	[61]
Black and green tea (*Camellia sinensis* L.)	Uncured pork sausages	Antioxidant activity on par with the effect of BHT without any adverse effects on sensory attributes	[62]
Cinnamon and clove	Baked foods	Growth inhibition of molds (*Aspergillus* spp., *Penicilium* spp.)	[63]
Onion and cranberry	Rabbit meat	Improve the microbial control against *Pseudomonas* and *Enterobacteriaceae*. Higher phenolic content	[64]
Stinging nettle leaves	Bread	Improve the antioxidant activity (DPPH), quality, and sensory profile	[65]
Olive (*Olea europaea* L.) leaves	Baked snacks	Reduction of oxidative degradation. Improvement of sensory data, antioxidant activity, and level of volatile compounds	[66]

**Table 2:** Examples of food applications of edible films and coatings based on different edible materials.

Coating Material	Product	Properties	Ref.
***Polysaccharides***			
Chitosan-based	Baby carrots	Delay of the microbial spoilage. Improvement of quality attributes: Product color and texture	[77]
Chitosan-green tea extract	Walnut kernel	Inhibition of lipid oxidation and fungal growth and optimization of sensory properties	[78]
Chitosan-gelatin	Beef	Improvement of color preservation and reduced weight loss and lipid oxidation	[79]
Chitosan-gelatin	White shrimp *(Litopenaeus vannamei)*	Shelf-life extension by decreasing the total and psychrotrophic bacteria. Decrease of lipid oxidation and improvement of texture and color	[80]
Chitosan-cassava starch-*Myrcia ovata* Cambessedes EO	Mangaba fruits	Growth inhibition of *Bacillus cereus, Bacillus subtilis*, and *Serratia marcescens*	[81]
Chitosan-pomegranate peel extract	White shrimp (*Litopenaeus vannamei)*	Retarding melanosis and color changes, enhancing texture and sensory scores during iced storage	[82]
Chitosan-whey protein	Ricotta cheese	Extension of shelf-life: Retard development of undesirable acidity and microbial spoilage without modifying sensory characteristics	[83]
Chitosan-lemongrass oil	Grape berry	Initial inhibition of *Salmonella typhimurium* and other microorganisms. Retention of color, total soluble solid content, and improvement of the antioxidant activity during storage	[84]
Chitosan clove oil	Cooked pork sausages	Inhibition of microbial growth, late lipid oxidation, and extension of the shelf-life in refrigerated storage	[85]
Chitosan-gallic acid	Pacific mackerel fillets	Synergistic effect during chilled storage: Inhibition of microbial growth, BA formation, lipid oxidation, and nucleotide and protein breakdown, without losing optimal sensory characteristics	[86]
Cellulose derivatives	Fried potatoes	Oil uptake reduction without causing differences in texture	[87]
Cellulose derivatives	Fresh eggs	Lower weight loss and improvement of albumen quality, changing from grade AA to A, and remaining that way during the storage period	[88]
Carboxymethyl cellulose (CMC)-chitosan	Citrus fruits	Improvement of fruit qualities: Firmness, weight loss, fruit gloss, fruit ripening progression, sensory evaluation, ethanol concentration, and disease incidence	[89]
Tapioca starch-based	Fortified pumpkin	Color, antimicrobial activity, and ascorbic acid retention were improved.	[90]
Starch-based	Brussels sprouts	Shelf-life extension by optimizing weight loss, surface color and texture	[91]
Starch-gelatin	Red Crimson grapes	Increase of biofilm mechanical strength, solubility in water, water vapor permeability, and thickness	[92]
Pectin-based	Fresh-cut carrots	Lower accumulation of phenolic acids (responsible for white blush) and flavonoids (responsible for astringency and bitterness) during refrigerated storage	[93]
Pectin-green tea powder	Pork patty	Decrease of lipid oxidation, increase of radical scavenging effects, and reduction of total aerobic bacteria	[94]
Pectin-oregano EO-resveratrol	Fresh pork loin	Decrease of pH, color change, lipid and protein oxidation, and inhibition of microbial growth with high oxygen modified atmosphere packaging	[95]
Agar-alginate-chitosan-AgNP-Grapefruit seed PE	Potatoes	UV-screening effect, antimicrobial activity against *Listeria monocytogenes,* and *Escherichia coli*. Prevention of greening during storage and formation of condensed water on the packaged film surface	[96]
Sodium alginate with anti-browning agents	Fresh-cut apple	Improvement of the shelf-life by reducing microbial growth and reducing the browning index	[97]
Sodium alginate-EO	*Arbutus unedo* L. Fresh fruit	Maintenance of postharvest quality (sensory and nutritional) attributes through storage. Reduction of microbial spoilage	[98]
Sodium alginate-CMC- epigallocatechin gallate	Fresh pork	Significant inhibitory effect on microbial growth, lipid oxidation, and improvement of sensory scores	[99]
K-carrageenan	Fortified pumpkin	Color, antimicrobial activity, and ascorbic acid retention were improved.	[90]
Pullulan	‘Fuji’ apples	Extended shelf-life. Retarding enzymatic browning, adecrease of weight loss, inhibition of microbial growth and maintenance of firmness	[100]
Xanthan gum-enriched with cinnamic acid	Fresh-cut pears	Prevention of browning, lower polyphenol oxidase activity, and extension of the shelf-life	[101]
***Proteins***			
Gelatin-tea polyphenol	Golden pomfret fillets	Reduction of the weight loss, pH lowering, and microbial growth inhibition. Retarding myofibril degradation during cold storage	[102]
Gelatin-chitosan	Golden pomfret fillets	Inhibition of myofibril degradation during cold storage	[103]
Gelatin-oregano EO	Rainbow trout fillets	Decrease of total volatile basic nitrogen, peroxide value, thiobarbituric acid, and microbial growth	[104]
Gelatin-CMC-chitin nanofibers-*Trachyspermum ammi* EO (Ajowan)	Refrigerated raw beef	Antimicrobial effect against psychrotrophic bacteria, *Pseudomonas* spp., *Staphylococcus aureus*, LAB, molds, and yeasts. Maintenance of chemical profile, color, and sensory properties	[105]
Whey protein	Frozen Atlantic salmon	Decrease of lipid oxidation of fish fillets. Increase of whiteness of cooked samples	[106]
Whey protein-oregano or clove EO	Chicken breast fillets	Antimicrobial effect against aerobic mesophilic bacteria, *Enterobacteriaceae*, total aerobic psychrotrophic bacteria, LAB, and *Pseudomonas* spp.	[107]
Whey protein-lactoperoxidase system-α-tocopherol	Pike-perch fillets	Antibacterial and antioxidant properties directed towards shelf-life extension	[108]
***Lipids***			
Candelilla wax	Strawberry	Antifungal activity against *Rhizopus stolonifera* and extension of postharvest shelf-life	[109]
Candelixa wax-ellagic acid	Avocado	Antimicrobial activity against *Colletotrichum gloeosporioides* and improvement of quality and shelf-life	[110]
Carnauba wax-cassava starch	Fresh-cut apples	Positive impact on water solubility and respiration rate. Improve film mechanical properties	[111]
Shellac and *Aloe vera* gel (resins)	Tomatoes	Improved permeability of O_2_, CO_2_, and water vapor. Senescence delay and shelf-life extension	[112]

**Table 3:** The main characteristics of spectroscopic techniques.

Spectroscopic Technique	Wavelength Limits	Type of Transition	Advantages	Limitations
**Fluorescence**	250–750 nm	Bonding electrons in molecules	Rapid, high accuracy, sensitivity, relatively low cost	Limited to samples containing fluorophores, sample surface technique
**Visible**	380–750 nm	Bonding electrons in molecules	Accuracy, sensitivity, relatively low cost, suitable to measure color	Effect of scattering and path length
**Near-infrared**	750–2500 nm	Overtones and combinations of fundamental bands	Less sample preparation requirement, high sensitivity to physical structure, and presence of water	Requires reliable reference methods, low specificity, overlapped and complex spectra, dry samples preferred
**Hyperspectral imaging**	400–1000 nm (Most common)	-	Providing spatial information (pixel-to-pixel signal)	Huge amount of data and data processing, costs
**Mid-infrared**	2500–25000 nm	Fundamental stretching, bending, and rotating	High sensitivity to chemical compositions, distinct absorption peaks	Water interference and low light penetration
**Raman**	750–1064 nm	Vibrational transitions	Provides structural and qualitative information, low sensitivity to water	Interference from biological fluorescence background signals, a small part of the sample is irradiated (laser spot), low sensitivity, due to weak scattering
**Nuclear magnetic resonance**	1–1000 m	Nuclei orientation into a magnetic field	Accuracy, determination of precise structures, minimal sample preparation, spatial information (magnetic resonance imaging: MRI)	Expensive equipment, low sensitivity, overlapping signal, especially when analyzing complex mixtures
